# Multisite Cannulation for Venovenous Extracorporeal Membrane Oxygenation Support in Neonates and Infants: Extracorporeal Life Support Database Study, 2013–2023

**DOI:** 10.1097/PCC.0000000000004009

**Published:** 2026-07-02

**Authors:** Jon Lillie, Shane M. Tibby, Peter Rycus, Marc Anders, Gail Annich, Matteo Di Nardo, Geoff Burnhill

**Affiliations:** 1 Paediatric Intensive Care Evelina London Children’s Hospital, London, United Kingdom.; 2 King’s College, London, United Kingdom.; 3 Extracorporeal Life Support Organisation (ELSO), Ann Arbor, MI.; 4 Department of Pediatrics, Section of Critical Care, Baylor College of Medicine, Houston, TX.; 5 Department of Critical Care Medicine, The Hospital for Sick Children, Toronto, ON, Canada.; 6 Pediatric Intensive Care Unit, Children’s Hospital Bambino Gesù, IRCCS, Rome, Italy.

**Keywords:** adverse events, cannula, extracorporeal membrane oxygenation, multisite, respiratory failure, venovenous

## Abstract

**OBJECTIVES:**

We have evaluated the complications and outcomes of venovenous multisite (VVMS) cannulation for support with extracorporeal membrane oxygenation (ECMO) using the Extracorporeal Life Support Organization (ELSO) registry of neonates (≤ 28 d old) and infants (29–364 d old).

**DESIGN:**

Retrospective ELSO database cohort, comparing outcomes and complications associated with VVMS and venovenous dual lumen (VVDL) cannulation.

**PATIENTS:**

Patients younger than 1 year supported with VV ECMO.

**SETTING:**

ELSO data January 1, 2013 to December 31, 2023.

**INTERVENTIONS:**

None.

**MEASUREMENTS AND MAIN RESULTS:**

We identified 1403 neonates and 603 infants supported with VVDL, and 42 and 77, respectively, with VVMS. At 24 hours of ECMO we found median (interquartile range [IQR]) oxygen saturations (Sao_2_) were higher in the VVMS compared with the VVDL group: for neonates, 98% (IQR, 93–99%) vs. 94% (IQR, 89–97%); a *p* value of less than 0.01; and for infants, 94% (IQR, 91–97%) vs. 90% (IQR, 85–95%); a *p* value of less than 0.01. After adjustment for ventilatory parameters, VVMS was associated with higher mean (95% CI) Sao_2_ than VVDL: for neonates by 3.3% (95% CI, 1.0–5.6%); *p* = 0.005; and for infants by 3.3% (95% CI, 1.3–5.3%); *p* = 0.002. Higher Sao_2_ was also associated with higher flows rather than ventilator parameters. Median flows were higher in the VVMS group compared with VVDL for neonates (111 vs. 101 mL/kg/min) and infants (114 vs. 100 mL/kg/min); a *p* value of less than 0.01. In general, we failed to identify associations between support technique and mortality or complications. However, in infants, VVMS in comparison with VVDL was associated with fewer mechanical complications (23% vs. 37%; *p* = 0.02).

**CONCLUSIONS:**

Between 2013 and 2023, in neonatal and infant ECMO, VVMS as opposed to VVDL cannulation was associated with higher ECMO flows and Sao_2_ at 24-hour support. We failed to identify associations between support technique and complications or mortality, apart from lower mechanical complications in infants supported using VVMS.

**RESEARCH IN CONTEXT**

- Most neonates and infants with respiratory failure requiring extracorporeal membrane oxygenation (ECMO) are supported via venoarterial cannulation due to concerns with venovenous dual lumen (VVDL) cannulas or the safety of venovenous multisite (VVMS) cannulas in smaller patients.
- Recent literature about VVMS cannulation in neonates and infants is limited to small studies.
- Therefore, we used the Extracorporeal Life Support Organization (ELSO) multicenter international database, 2013–2023, to study the associated outcomes and complications of VVMS vs. VVDL in neonates and infants.

**AT THE BEDSIDE**

- In the ELSO registry, 2013– 2023, the use of VVMS has increased over time.
- In patients younger than 1 year, use of VVMS ECMO in comparison to VVDL was associated with higher Sao_2_ and flows at 24 hours of support. Infants supported via VVMS compared with VVDL also had an associated lower rate of mechanical complications.
- This retrospective analysis of the ELSO data shows that VVMS is used as an alternative cannulation strategy to VVDL in patients younger than 1 year.

Extracorporeal membrane oxygenation (ECMO) is an established support therapy for severe respiratory failure that is refractory to conventional mechanical ventilation. In adult practice, venovenous ECMO (VV ECMO) is the preferred form of support and has a conditional recommendation (with low evidence) in the 2024 American Thoracic Society updated practice guidelines (1). In contrast, in neonatal (≤ 28 d old) respiratory failure, according to the Extracorporeal Life Support Organization (ELSO) registry, use of VV ECMO as opposed to venoarterial ECMO (VA ECMO) reached a nadir of 8% in 2021, then rose to 22% in 2023 and 2024 (2). Regarding ECMO support in pediatric respiratory cases (> 28 d to < 18 years of age), in the ELSO registry for 2014–2024, there are over 5000 pediatric refractory respiratory failure patients that have been supported with VV ECMO (3), and among these, 8.1% were converted to VA ECMO; the latter modality being associated with significantly higher in-hospital mortality and greater prevalence of complications. The number of pediatric respiratory VV ECMO runs in the ELSO registry between 2019 and 2022 was around 500 per year, which represented around one-third to one-quarter of all pediatric ECMO support (4).

Cannulation for VV ECMO can be performed in two ways. First, using a venovenous dual lumen cannula (VVDL) at a single site, usually the right internal jugular (RIJ) vein. Second, by using two (or more) separate, single-lumen cannulas at multiple sites-VV multisite (VVMS): either internal jugular and femoral or, in older children, bi-femoral. When 11 ECMO centers in the United Kingdom were surveyed in 2018–2019, respondents identified concerns with dual lumen cannulation as the main reason for utilizing VA ECMO over VV ECMO in neonates (5). In particular, the difficulty in siting the cannulas, risk of cardiac/vessel perforation, and the potential for achieving insufficient flow rates. Historically, a review of ELSO data from 2015 to 2019 (6) as well as the ELSO Sixth Edition of the “Red Book” from 2022 (7) indicated that VVMS cannulation was to be avoided until children were ambulatory because of concerns that the femoral veins were too small in infants and children, potentially resulting in inadequate flows and/or neurovascular compromise. However, four recent pediatric studies—including single-center reports from 2015 to 2019 (8) and 2017 to 2021 (9), an ELSO registry study from 2000 to 2019 (10), and a three-center report from 2017 to 2024 (11)—each describe VVMS (internal jugular and femoral) cannulation in patients as small as 1.8 kg and show that adequate flows and oxygenation can occur without clinical limb compromise. We therefore aimed to compare the VVMS and the VVDL cannulation strategies in neonatal and infant cases using the ELSO registry dataset for 2013–2023. Our primary aim was to compare oxygen saturation and ECMO flow at 24 hours in the two cannulation strategies. The secondary aims were to compare the associated prevalence of complications and mortality by cannulation strategy.

## METHODS

This ELSO registry project was approved by the Scientific Oversight Committee of ELSO in 2022. Local approval was also obtained from Evelina London’s Service Evaluation team, which waived individual consent because all data were fully anonymized (a process that is equivalent to an Institutional Review Board) on November 3, 2022 (Project No. 11834). All handling of ELSO registry patient data was undertaken in accordance with local committee requirements and fully compliant with the ethical standards of the Helsinki Declaration of 1975, amended 2024.

The ELSO database was interrogated to extract all VV ECMO runs in patients younger than 1 year over the 11-year period, from January 1, 2013 to December 31, 2023. Neonates were defined as 28 days old or younger and infants as 29–364 days of age at cannulation. Data collected included year of ECMO, demographics, diagnoses, pre-ECMO clinical status, cannulation mode, status on ECMO at 4 and 24 hours postcannulation (including flow rates achieved, arterial oxygen-hemoglobin saturation [Sao_2_ and ventilatory settings], complications, and survival to discharge from the ECMO center).

Patients were excluded if weight was the greater than 99.999^th^ centile for age or cannulation details were absent. Those in whom a definitive initial cannula configuration could not be identified were excluded, which incorporated patients who received VA ECMO and VV ECMO and those receiving both VVMS and VVDL. Finally, patients in whom ECMO flows were absent or unfeasible (e.g., > 500 mL/kg/min) were excluded (**Fig. S1**, https://links.lww.com/PCC/C756). Cannula sizes were grouped into French (Fr) size ranges (“bands”) to maintain manufacturer anonymity, for example, 7–8-Fr or 13–14-Fr. We could not differentiate between VVDL cannulas designed for mid-atrial placement (e.g., Crescent) and those intended for bicaval positioning in the inferior vena cava (e.g., Avalon).

### Statistical Analysis

The two coprimary outcomes were prespecified before the data analysis. The first was Sao_2_ at 24-hours post-ECMO commencement, with saturations expressed as both continuous and a binary variable using the threshold of “success” as oxygen saturations greater than or equal to 85%, defined a priori using the 2012 ELSO “Red Book” Fourth Edition (12). The second coprimary endpoint was ECMO flow at 24 hours. The secondary endpoints also included ECMO-related complications and in-hospital mortality as described by ELSO in its 2025 data definitions (13).

Neonatal and infant data were analyzed separately. In all analyses, a *p* value of less than 0.05 was considered significant. No corrections have been made to the *p* value for multiple comparisons. For the multivariable models, potential confounders were identified a priori, based upon previous literature, and differed according to the outcome, age group, and modeling used (see individual model results). For mortality these were weight, cardiac arrest, pH, and oxygenation index (OI). The additional variables used in the neonatal analysis included gestational age and diagnosis of congenital diaphragmatic hernia (CDH).

Bivariate comparisons between VVMS and VVDL were undertaken via Student *t* test Wilcoxon rank-sum, and chi-square tests, as appropriate. Data are summarized as median and interquartile range (IQR) or proportions with percentages when the denominator of the percentage is greater than 50. Differences in proportions, when notable, are presented with a difference in percentage and the 95% CI of the difference. Comparison of change in Sao_2_ and ECMO flow over time (coprimary outcomes) used a hierarchical linear mixed-effects model with random intercepts per patient, estimated via restricted maximum likelihood, to model the change in each variable over time for the two cannula groups (VVMS and VVDL). This process allows for missing data (e.g., if a patient did not have measurements made at both time points). Each analysis provides three *p* values per outcome variable: 1) “time,” representing an overall change in the outcome over time, regardless of cannula group; 2) “group,” representing a systematic difference between the cannula groups across time; and 3) “interaction,” estimating whether the difference in the outcome variable varied across time between the two groups.

The association between cannula type and Sao_2_ at 24 hours after establishing ECMO was also modeled using multivariable linear regression, which included adjustment for the confounding effects of ventilatory settings at this time point. Other factors associated with mortality were modeled using multivariable logistic regression. Both regression methods also incorporated multiple imputation (m = 10 imputations), under the assumption that data were missing completely at random and that missingness was less than 50%; the former was checked by comparing non-missing variables in each group for each chosen outcome. Results of regression analyses are presented with adjusted odds ratio (aOR) and 95% CI.

Initial data cleaning was performed using Microsoft Excel (2019, Microsoft Corporation, Redmond, WA). Further data cleaning and simple bivariate comparisons used *R Studio* (12.0 + 467, 2024, PBC, Boston, MA). Multivariable modeling was performed using Stata Version 15.1 (StataCorp, College Station, TX).

## RESULTS

Over the 11 years, 2013–2023, there were 3111 VV ECMO runs identified in the registry for patients younger than 1 year of age. After exclusions, there were 1445 VV runs in neonates and 680 in infants, respectively (Fig. S1, https://links.lww.com/PCC/C756). Overall, the proportion of venovenous runs delivered as VVMS was 3% (42/1445) for neonates across 14 centers, and 11% (77/680) for infants (**Tables 1** and **2**).

**TABLE 1.** Comparison of Preextracorporeal Membrane Oxygenation Characteristics for Neonatal Runs

Neonates (*n* = 1445)			
Variable	Venovenous Dual Lumen, *n* (%)	Venovenous Multisite, *n*/*n*	*p*
*n*	1403	42	
Weight, kg, median (IQR)	3.4 (3.0–3.8)	3.3 (2.7–3.5)	0.02
Gestation, wk, median (IQR)	39 (38–40)	39 (37–40)	0.95
Age, d, median (IQR)	1 (1–3)	2 (1–4)	0.56
Cardiac arrest	93 (7)	3/42	0.90
Diagnosis			
Persistent pulmonary hypertension of the newborn	708 (50)	19/42	0.5
Meconium aspiration syndrome	639(46)	15/42	0.20
Acute respiratory distress syndrome	319 (23)	7/42	0.35
Congenital diaphragmatic hernia	196 (14)	6/42	1
pH (IQR)	7.18 (7.07–7.28)	7.15 (7.04–7.21)	0.04
Pco_2_, mm Hg, median (IQR)	60 (48–77)	66 (50–81)	0.33
Po_2_, mm Hg, median (IQR)	38 (28–48)	37 (30–45)	0.79
Hco_3_, mmol/L, median (IQR)	22.3 (20–25)	19.7(18–22)	< 0.01
Sao_2_, %, median (IQR)	70 (47–84)	70 (56–81)	0.94
Fio_2_, %, median (IQR)	100 (100–100)	100 (100–100)	0.12
Peak inspiratory pressure, cm H_2_O, median (IQR)	35 (28–44)	30 (25–35)	< 0.01
Peak end-expiratory pressure, cm H_2_O, median (IQR)	7 (6–9)	6 (5–8)	0.21
Mean airway pressure, cm H_2_O, median (IQR)	18 (15–20)	16 (12–18)	0.03
Oxygenation index, median (IQR)	47 (33–65)	42 (34–64)	0.61

IQR = interquartile range.

Median (IQR) is shown for continuous data, or *n* (percentage) or proportion is shown if *n* < 50 for categorical data. The Wilcoxen rank-sum test, χ^2^ test, and Student *t* test were used as appropriate. Univariable analyses were undertaken without adjusting for multiple comparisons. Patients may have more than one diagnosis.

**TABLE 2.** Comparison of Preextracorporeal Membrane Oxygenation Characteristics of Infants

Infants (*n* = 680)			
Variable	Venovenous Dual Lumen	Venovenous Multisite	*p*
*n*	603	77	
Weight, kg, median (IQR)	5.5 (4.2–7.0)	5.8 (4.4–7.7)	0.51
Age, d, median (IQR)	152 (76–229)	172 (97–263)	0.04
Cardiac arrest, *n* (%)	51 (8)	7 (9)	0.88
Diagnosis, *n* (%)			
Acute respiratory distress syndrome	385 (64)	48 (62)	0.9
Viral	177 (29)	17 (22)	0.23
Sepsis	74 (12)	8 (10)	0.77
Bacterial	62 (10)	7 (9)	0.9
pH, median (IQR)	7.26 (7.14–7.37)	7.24 (7.11–7.37)	0.38
Pco_2_, mm Hg, median (IQR)	67 (52–88)	66 (50–85)	0.74
Po_2_, mm Hg, median (IQR)	53 (40–70)	44 (34–67)	0.02
Hco_3_, mmol/L, median (IQR)	30 (25–35)	27 (23–32)	0.03
Sao_2_, %, median (IQR)	84 (74–92)	82 (65–90)	0.12
Fio_2_, %, median (IQR)	100 (96–100)	100 (100–100)	0.22
Peak inspiratory pressure, cm H_2_O, median (IQR)	35 (28–42)	30 (25–38)	<0.01
Peak end-expiratory pressure, cm H_2_O, median (IQR)	9 (6–11)	10 (6–12)	0.78
Mean airway pressure, cm H_2_O, median (IQR)	21 (17–25)	19 (15–22)	0.03
Oxygenation index, median (IQR)	37 (23–52)	38 (30–52)	0.43

IQR = interquartile range.

Median (IQR) is shown for continuous data, or *n* (%) for categorical data. The Wilcoxen rank-sum test, χ^2^ test, and Student *t* test were used as appropriate. Univariable analyses were undertaken without adjusting for multiple comparisons. Patients may have more than one diagnosis.

The use of VVMS increased in 2023 in comparison with the overall values, to 13% (12/95) in neonatal ECMO runs and 22% (10/45) in infant ECMO runs (**Fig. S2**, https://links.lww.com/PCC/C756). Cannula size was recorded for both cannulas in 34 of 42 neonatal runs receiving VVMS with variability in the combination of cannulas chosen, particularly for the access cannula in the RIJ, which ranged from 8 to 14 Fr (**Table S1**, https://links.lww.com/PCC/C756). For the femoral vein in neonatal runs 24 of 34 had a 7-/8-Fr return cannula. For VVDL neonatal runs, cannula size was recorded in 1400 of 1403, of which 71% (1034/1400) had a 13-/14-Fr cannula.

### Demographics and Pre-ECMO Variables

On comparison of ECMO runs in neonates, by support mode (VVMS vs. VVDL), VVMS is associated with smaller median (IQR) patient weight: 3.3 kg (IQR, 2.7–3.5 kg) vs. 3.4 kg (IQR, 3.0–3.8 kg); *p* = 0.02 (Table 1). Regarding the blood gas and oxygenation data we found that the VVMS vs. VVDL grouping showed an associated lower median pH before ECMO (7.15 [IQR, 7.04–7.21] vs. 7.18 [IQR, 7.07–7.28]; *p* = 0.04), but we failed to identify any other associations. In the ventilatory parameters, pre-ECMO VVMS as opposed to VVDL, was associated with lower median levels of peak inspiratory pressure (PIP; 30 cm H_2_O [IQR, 25–35 cm H_2_O] vs. 35 cm H_2_O [IQR, 28–44 cm H_2_O]; *p* < 0.01), and we failed to identify any associations with positive end-expiratory pressure (PEEP) level or Fio_2_.

A comparison of the ECMO runs in infants is summarized in Table 2. By group, support with VVMS as opposed to VVDL was associated with older median age (172 d [IQR, 97–263 d] vs. 152 d [IQR, 76–229 d]; *p* = 0.04). On other testing we identified an association between grouping (VVMS vs. VVDL) and lower pre-ECMO median Pao_2_: 44 mm Hg (IQR, 34–67 mm Hg) vs. 53 mm Hg (IQR, 40–70 mm Hg); *p* = 0.02. There were also associations between grouping and mean airway pressure (MAP) and PIP, with lower median values identified in the VVMS vs. VVDL groups, respectively: 19 cm H_2_O (IQR, 15–22 cm H_2_O) vs. 21 cm H_2_O (IQR, 17–25 cm H_2_O); *p* = 0.03; and 30 cm H_2_O (IQR, 25–35 cm H_2_O) vs. 35 cm H_2_O (IQR, 28–42 cm H_2_O); *p* value of less than 0.01.

### Outcomes

The unadjusted, coprimary outcomes of Sao_2_ and ECMO flows, along with blood gas and ventilatory variables at 24 hours postcannulation, are shown in **Table 3**. For neonates, there was an association between support grouping (VVMS vs. VVDL) and higher median Sao_2_ in VVMS: 98% (IQR, 93–98%) vs. 94% (IQR, 89–97%); *p* value of less than 0.01; we failed to identify an association between support group and the proportion of runs with Sao_2_ greater than 85% at 24 hours of ECMO. In the linear mixed model-adjusted trajectories for both primary outcome variables, we failed to identify an association between grouping and overall increase in Sao_2_ at 24 hours postcannulation (group *p* = 0.26; interaction *p* = 0.77; see **Fig. S3**, https://links.lww.com/PCC/C756, top left https://links.lww.com/PCC/C756) with the confounder-adjusted association between cannula type and Sao_2_ at 24 hours shown in **Table 4**. After adjusting for baseline Sao_2_, and both Fio_2_ and PIP at 24 hours, neonatal support with VVMS was associated with mean Sao_2_ approximately 3.3% (95% CI, 1.0–5.6%) higher than those supported with VVDL (*p* = 0.005). This finding may have been because neonatal VVMS is associated with a better increment in flow than VVDL (interaction *p* = 0.03; see Fig. S3, top right, https://links.lww.com/PCC/C756). The adjusted, 24-hour mean ECMO flows for VVMS vs. VVDL were, respectively, 115 mL/kg/min (95% CI 106–124 mL/kg/min) vs. VVDL, 105 mL/kg/min (95% CI, 104–107 mL/kg/min). Note that out of MAP, PEEP, and PIP, only PIP was of influence in this model.

**TABLE 3.** Differences Between Groups on Extracorporeal Membrane Oxygenation With Parameters at 24 hr of Extracorporeal Membrane Oxygenation Shown

On ECMO Parameters: Neonates			
Variable	Venovenous Dual Lumen	Venovenous Multisite	*p*
*n*	1403	42	
pH, median (IQR)	7.37 (7.33–7.42)	7.35 (7.28–7.41)	0.06
Pco_2_, mm Hg, median (IQR)	44 (39–49)	45 (39–50)	0.80
Po_2_, mm Hg, median (IQR)	65 (54–83)	77 (60–99)	0.04
Hco_3_, mmol/L, median (IQR)	25 (23–28)	23 (19–27)	0.02
Sao_2_, %, median (IQR)	94 (89–97)	98 (93–99)	< 0.01
Sao_2_ ≥ 85%, % (*n*/*n*)	87 (1117/1277)	36/38	0.13
Fio_2_, %, median (IQR)	40 (30–50)	52 (30–76)	< 0.01
PIP, cm H_2_O, median (IQR)	20 (20–23)	20 (18–22)	0.23
PEEP, cm H_2_O, median (IQR)	8 (6–10)	7 (5–10)	< 0.01
Mean airway pressure, cm H_2_O, median (IQR)	11 (10–13)	10 (7–12)	< 0.01
ECMO Flow ml/kg/min, median (IQR)	101 (90–116)	111 (99–131)	< 0.01
Length of ECMO run, median (IQR)	121 (88–195)	137 (82–227)	0.69
Survival, *n* (%)	1118 (80)	32/42	0.72

On ECMO Parameters: Infants			
*n*	603	77	
pH, median (IQR)	7.39 (7.35–7.44)	7.39 (7.34–7.47)	0.93
Pco_2_, mm Hg, median (IQR)	48 (42–54)	45 (40–51)	0.04
Po_2_, mm Hg, median (IQR)	59 (50–72)	62 (52–80)	0.2
Hco_3_, mmol/L, median (IQR)	29 (25–33)	27 (24–30)	0.01
Sao_2_, %, median (IQR)	90 (85–95)	94 (91–97)	< 0.01
Sao_2_ ≥85%, % (*n*/*n*)	76 (424/555)	87 (60/69)	0.04
Fio_2_, %, median (IQR)	40 (40–60)	50 (40–100)	< 0.01
PIP, cm H_2_O, median (IQR)	21 (20–25)	23 (20–26)	0.25
PEEP, cm H_2_O, median (IQR)	10 (8–10)	10 (7–10)	0.45
Mean airway pressure, cm H_2_O, median (IQR)	13 (11–15)	13 (11–15)	0.89
ECMO flow, mL/kg/min, median (IQR)	100 (89–115)	114 (97–138)	< 0.01
Length of ECMO run, median (IQR)	209 (124–347)	181 (95–336)	0.15
Survival, *n* (%)	438 (73)	55 (71)	0.93

ECMO = Extracorporeal Membrane Oxygenation, IQR = interquartile range, PEEP = peak end-expiratory pressure, PIP = peak inspiratory pressure.

Median (IQR) is shown for continuous data, or *n* (%) or proportion is shown if *n* < 50 for categorical data. The Wilcoxen rank-sum test, χ^2^ test, and Student *t* test were used as appropriate. Univariable analyses were undertaken without adjusting for multiple comparisons.

Survival is counted as discharged alive, off ECMO, from center.

**TABLE 4.** Multivariable Linear Regression Models Examining Factors Associated With Sao_2_ 24 hr Postextracorporeal Membrane Oxygenation Initiation

Neonates (*n* = 1315)				
Variable	% Missing	Coefficient	95% CI	*p*
VVMS	0	3.3	(1.02–5.6)	0.005
Sao_2_ % at ECMO initiation	7.5	0.04	(0.03–0.06)	< 0.001
Fio_2_ % at 24 hr	2.4	–0.02	(–0.04 to –0.002)	0.03
PIP (cm H_2_O) at 24 hr	6.9	–0.08	(–0.16 to 0.01)	0.09

Infants (*n* = 624)				
Variable	% Missing	Coefficient	95% CI	*p*
VVMS	0	3.3	(1.3–5.3)	0.001
Sao_2_ % at ECMO initiation	12.0	0.09	(0.06–0.13)	< 0.001
Fio_2_ % at 24 hr	2.9	0.01	(–0.02 to 0.04)	0.52
PIP (cm H_2_O) at 24 hr	8.7	–0.001	(–0.13 to 0.13)	0.98

ECMO = extracorporeal membrane oxygenation, PIP = peak inspiratory pressure, VVMS = venovenous multisite.

Missing data were accounted for using multiple imputation. Note that the total numbers of neonates and infants were slightly reduced due to missing oxygen saturations at 24 hr, which could not be imputed.

For infants, VVMS grouping as opposed to VVDL was associated with higher median Sao_2_: 94% (IQR, 91–97%) vs. 90% (IQR, 85–95%); *p* value of less than 0.01. Grouping (VVMS vs. VVDL) was also associated with a greater proportion of infants with “successful” Sao_2_ (i.e., ≥ 85%): 60 of 69 (87%) vs. 424 of 555 (76%); mean percentage difference 11% (95% CI, 0.5–18.1%); *p* = 0.04 (Table 3). Grouping (VVMS vs. VVDL) was also associated with greater improvement in Sao_2_ 24 hours postcannulation (interaction, *p* = 0.003; see Fig. S3, bottom left panel https://links.lww.com/PCC/C756). Also, like the observation in neonatal runs, after adjusting for confounders, the VVMS in comparison with VVDL was associated with increased mean 24-hour Sao_2_: difference 3.3% (95% CI, 1.3–5.3%); *p* = 0.002 (Table 4). As with neonatal runs, this increment was also associated with greater improvement in 24-hour ECMO flows for VVMS patients (interaction, *p* = 0.002; see Fig. S3, bottom right panel https://links.lww.com/PCC/C756). The adjusted, infant 24-hour mean ECMO flows for VVMS vs. VVDL were, respectively: 121 mL/kg/min (95% CI, 113–129 mL/kg/min) vs. 104 mL/kg/min (101–107 mL/kg/min).

Overall, we failed to identify an association between cannulation technique and risk of mortality in both neonate and infant groups (**Table S2**, https://links.lww.com/PCC/C756). In the multivariable analysis for neonates, we failed to identify whether VVMS compared with VVDL was associated with greater or lesser odds of mortality: aOR, 1.06 (95% CI, 0.49–2.30); *p* = 0.88. For infants, the corresponding analysis showed aOR, 1.05 (95% CI, 0.60–1.81); *p* = 0.87.

**Table 5** summarizes the complications data by age group and cannulation type. In general, we failed to find an association between cannulation type and complications, by age. However, in the infants, there was an association between cannula type (VVDL vs. VVMS) and a greater proportion of runs having a mechanical complication, respectively: 228 of 603 (37%) vs. 18 of 77 (23%); mean percentage difference 14% (95% CI, 2.8–22.9%); *p* = 0.02. Even so, we failed to identify an association with cannula problems (VVDL vs. VVMS), respectively: 81 of 603 (13%) vs. 5 of 77 (6%); mean percentage difference 6.9% (95% CI, –1.3% to 11.6%); *p* = 0.09.

**TABLE 5.** Extracorporeal Membrane Oxygenation-Associated Complications as Defined in the Methods Section

Neonates (*n* = 1445)			
Complications	VVDL, *n* (%)	VVMS, *n*/*n*	*p*
*n*	1403	42	
Any complication	785 (56)	20/42	0.36
Mechanical	420 (30)	12/42	0.98
Cannula problems	143 (10)	1/42	0.16
Cardiac	70 (5)	0	0.26
Pulmonary	67 (5)	4/42	0.29
Neuro	95 (7)	3/42	1.00
Metabolic	142 (10)	5/42	0.91
Hemorrhagic	61 (4)	3/42	0.63
Cannulation site	15 (1)	0	1.0
Renal	334 (24)	11/42	0.86
Infection	5 (0)	0	1.00
Tamponade	22 (2)	0	0.86
Limb	3 (0)	0	1.00
Survival	1118 (80)	32/42	0.72

Infants (*n* = 680)			
Complications	VVDL, *n* (%)	VVMS, *n* (%)	*p*
*n*	603	77	
Any complication	356 (59)	41 (53)	0.40
Mechanical	228 (37)	18 (23)	0.02
Cannula problems	81 (13)	5 (6)	0.09
Cardiac	45 (7)	4 (5)	0.62
Pulmonary	51 (8)	4 (5)	0.44
Neuro	27 (4)	6 (8)	0.32
Metabolic	79 (13)	4 (5)	0.07
Hemorrhagic	42 (7)	8 (10)	0.39
Cannulation site	13(2)	0	0.39
Renal	149 (25)	15(19)	0.38
Infection	4 (1)	0	1.00
Tamponade	9 (1)	1 (1)	1.00
Limb	4 (1)	0	1.00
Survival	438 (73)	55 (71)	0.93

VVDL = venovenous dual lumen, VVMS = venovenous multisite.

For categorical data: *n* (%) or proportion shown if *n* < 50. The Wilcoxen rank-sum test, χ^2^ test, and Student *t* test were used as appropriate. Univariable analyses were undertaken without adjusting for multiple comparisons.

Mechanical complications are: Air in the circuit, cannula problems, circuit change, clots and air emboli, clots:hemofilter, other tubing rupture, oxygenator failure, pump failure, raceway rupture, temperature device malfunction, and thrombosis/clots circuit component; there were no statistically significant differences within these subcategories.

## DISCUSSION

In this retrospective study of data held within the ELSO registry, 2013–2023, we have compared VVMS and VVDL ECMO runs in neonates and infants. The data show that ECMO support using VVMS was associated with higher Sao_2_ at 24 hours of ECMO and higher ECMO flows. The difference in Sao_2_ between the VVMS and VVDL groups remained significant after adjusting for confounders of pre-ECMO Sao_2_, ventilator Fio_2_ and PIP. In general, we failed to identify any association between cannulation technique and ECMO complications or mortality. However, in infants, we did observe an association between cannulation technique and a lower proportion of mechanical complications. Overall, during 2013–2023, the use of VVMS was small (i.e., 42 runs in neonates and 77 runs in infants). However, in 2023 there were 12 neonates and 10 infants supported with VVMS, which was proportionately higher than the average for the whole period.

In VV ECMO, Sao_2_ is primarily determined by pulmonary oxygen uptake and the proportion of venous return delivered to the oxygenator—unless significant recirculation occurs. Our analysis suggests that the improved Sao_2_ in the VVMS group was associated with cannulation mode itself rather than confounders such as ventilator Fio_2_, pressures on the ventilator or higher pre-ECMO saturations. The higher Fio_2_ could be due to variation in center practices or the need to deliver a higher Fio_2_ in the native lung as ECMO was not meeting the demands of the patient. This explanation is suggested in the neonatal group by higher Fio_2_ being negatively associated with higher Sao_2_ at 24 hours (Table 4). The most likely mechanism for higher Sao_2_ in VVMS runs was that this mode was associated with higher flows than VVDL, but the ELSO database does not contain the data required to explore this further.

The higher flows associated with VVMS compared with VVDL have been described in three single-center studies of neonates and infants, including a 2015–2019 study in 11 infants weighing less than 10 kg (8); a 2017–2021 study in 54 neonates (9); and a 2021–2023 study in 58 neonates and infants (< 1yr) (14). Patients from two of these studies (8, 14) were entered into the ELSO registry for the 2013–2023 period that we have studied, so our work essentially provides a more contextual analysis across several centers. Taken together, it is logical that higher flows can be achieved with VVMS compared with VVDL. The dimensions of the individual lumens comprising VVDL cannulas are not published but are smaller than the respective single-lumen cannulas used for VVMS for a given weight or size. Additionally, single-lumen cannulas can be positioned independently, whereas the fixed distance between the drainage and return ports in VVDL cannulas limits their adjustability, with the largest case series of the mid-atrial Crescent cannula reporting flow issues in a third of patients (14, 15). Higher flows and stability of flows are a potential benefit of VVMS and could have significant benefits for smaller babies, including those born prematurely, where placing a 13-Fr into the RIJ is technically challenging (16). Managing patients with CDH has additional challenges, which may be better suited to VVMS. For example, the anatomical abnormalities in these babies include smaller neck vessels that may not be able to accommodate large cannulas (17). For neonates, the most used cannula combination in the ELSO registry data (2013–2023) was 10-Fr in the internal jugular and 8-Fr in the femoral vein (Table S2, https://links.lww.com/PCC/C756). A 2017–2024, three-center study of 58 neonatal and pediatric patients weighing less than 15 kg showed that this combination provided a median flow of close to 400 mL/min (11). (Again, some of these data will be in the 2013–2023 ELSO dataset that we have studied.) The ability to deliver effective flow with a more appropriately sized 10-Fr RIJ cannula could, therefore, allow more babies with CDH to receive VV ECMO (9, 11).

We failed to identify an association between cannulation technique and mortality. Some of the known risk factors for mortality, such as lower weight, lower gestation and CDH, were associated with mortality in our subset of the 2013–2023 ELSO registry, but the failure to identify other associations is likely a consequence of limited sample size and perhaps the need to control for already known associations with outcome. For example, we note that other larger studies using the ELSO database, with populations that overlap our cohort, have already looked at relationships between ECMO support and mortality: 1) early hypoxia or moderate hyperoxia after ECMO initiation and greater odds of mortality in neonates, 2015–2020 (18); 2) neonates with CDH and pre-ECMO mode of ventilation with greater odds of mortality associated with use of high-frequency oscillatory ventilation, 2009–2019 (19); and 3) relationship between neonatal gestational age and worse ECMO outcomes, 2007–2017 (20). Our addition to these is that we found a higher percentage of infants with mechanical complications in the VVDL compared with the VVMS group (37% vs. 23%), with the 95% CI of the difference 2.8–22.9%. That is, we cannot exclude the possibility that the difference between the two techniques could be greater by one-in-five cases. We failed to identify an association with cannula problems (VVDL vs. VVMS). However, on closer inspection of the mean difference and upper limit of the 95% CI of the difference in cannula problems, we cannot exclude the possibility that VVDL is associated with greater complications than VVMS by up to one-in-10 cases. Of note, cannula problems are defined by ELSO as those “requiring intervention for misplacement, dislodgement, clots/fibrin, mechanical failure, or inappropriate position,” so this difference is plausible and reflects concerns that VVDL may have flow issues and need repositioning (5, 14).

The trends in cannulation technique (Fig. S2, https://links.lww.com/PCC/C756) show that VVMS was infrequently used at younger ages, although its use was highest in 2023, the most recent year of the cohort studied. This increase is likely to be due to reports of using VVMS cannulation in neonates down to less than 2 kg bodyweight and ongoing concerns with VVDL cannulas (5, 8, 9, 11, 21). Although there is a new VVDL cannula available (Crescent), it is important to note that a 2021–2023 cohort of 58 neonates and infants (< 1 yr) reported issues with flows and the need for recurrent cannula manipulation with surgical reposition in 30% of the patients (14). Most neonates with respiratory failure are still placed on VA ECMO, and additional data for venovenous cannulation techniques provide an opportunity to change the landscape of ECMO support for respiratory failure. Taking all the above together, our ELSO cohort study (2013–2023) provides further evidence that VVMS is an alternative option to VVDL for neonates and infants.

Our study has some limitations. First, the degree of missing and conflicting data about cannulation in the ELSO data set is the biggest issue. It was difficult to assess the cannulation mode in 986 of 3111 (32%) of the VV ECMO runs because of inadequate documentation leading to exclusion in the current analysis. Second, as cannulas were not associated with a specific run but with the overall ECMO episode, it was not possible to evaluate patients who had modifications to cannulation strategy within one episode in our ELSO dataset. Although a study of ECMO runs for pediatric patients (ELSO, 2014–2024) showed a venovenous to venoarterial conversion rate of approximately 8% (421/5162 cases), the analysis was undertaken using the mode of ECMO rather than granular cannulation data, which our study required (3). This problem could be an important limitation because a key difference between VVMS and VVDL could be the need to change cannula configuration or convert to VA ECMO if there were inadequate flows or oxygenation. Third, it was not possible to fully explore why Sao_2_ was higher in the VVMS group compared with VVDL, given our limited sample size. Potential factors that were not studied include fraction of oxygen delivered by ECMO, recirculation, and ECMO shunt fraction. Fourth, the ELSO registry database does not distinguish between bicaval VVDL such as the Avalon and mid-atrial VVDLs such as the Crescent, which was approved by the U.S. Food and Drug Administration during our study period (2013–2023). There may be differences in Sao_2_ and flows delivered between different types of VVDL cannulas, but we could not assess these. Last, the ELSO registry does not contain longer-term outcomes such as vessel patency and deep vein thrombosis.

In conclusion, in the ELSO registry dataset for 2013–2024, support with VVMS as opposed to VVDL was associated with higher Sao_2_ and ECMO flows at 24 hours in both neonates (≤ 28 d) and infants (29 d to < 1 yr). In general, we failed to identify an association between cannulation technique and complications or mortality; however, there was an association between use of VVMS and lower mechanical complications. The time trends in ELSO data also show that practitioners are using VVMS as an alternative to VVDL in patients younger than 1 year of age.

## ACKNOWLEDGMENTS

We thank the extracorporeal membrane oxygenation registry of the Extracorporeal Life Support Organization (Ann Arbor, MI), particularly the scientific oversight committee, for their support and approval of this project.

## Supplementary Material
