# Supplementary material for: Multisite Cannulation for Venovenous Extracorporeal Membrane Oxygenation Support in Neonates and Infants: Extracorporeal Life Support Database Study, 2013–2023
Source: Pediatr Crit Care Med. 2026 Jul 2;27(10):1287–98. doi: 10.1097/PCC.0000000000004009 (PMC13641257; doi:10.1097/PCC.0000000000004009)
Supplement: Supplementary file 1 [file pcc-27-1287-s001.pdf]

SUPPLEMENTAL DIGITAL CONTENT

Figure S1. STROBE (Strengthening the Reporting of Observational Studies in Epidemiology) diagram showing total number of respiratory ECMO runs recorded in ELSO and reasons for excluding patients prior to analysis.

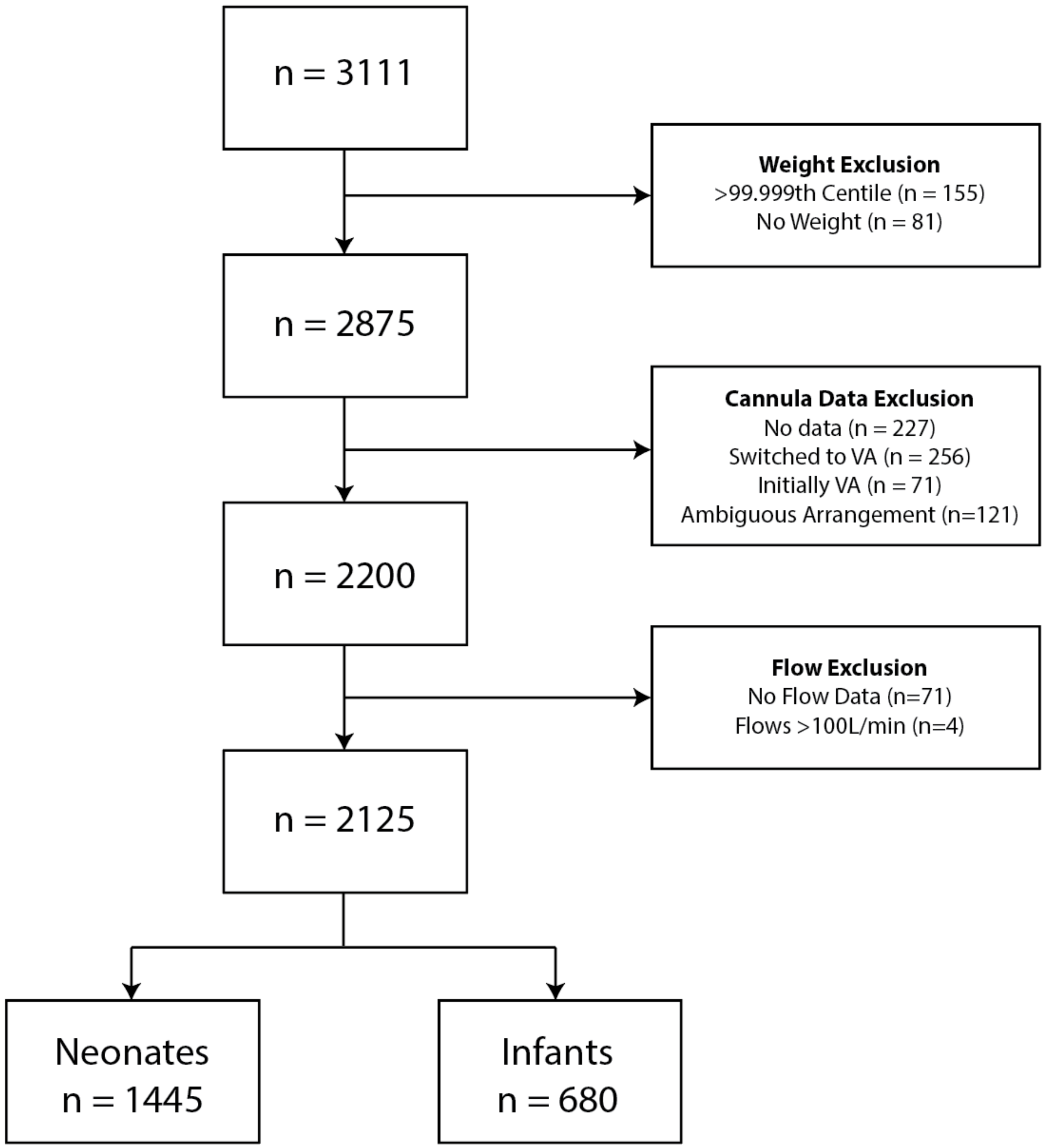

Figure S2. Charts showing number of ECMO runs by year divided by cannulation mode. DL: Dual lumen cannula. MS: multisite is where patient had two cannulas and neither recorded as dual lumen.

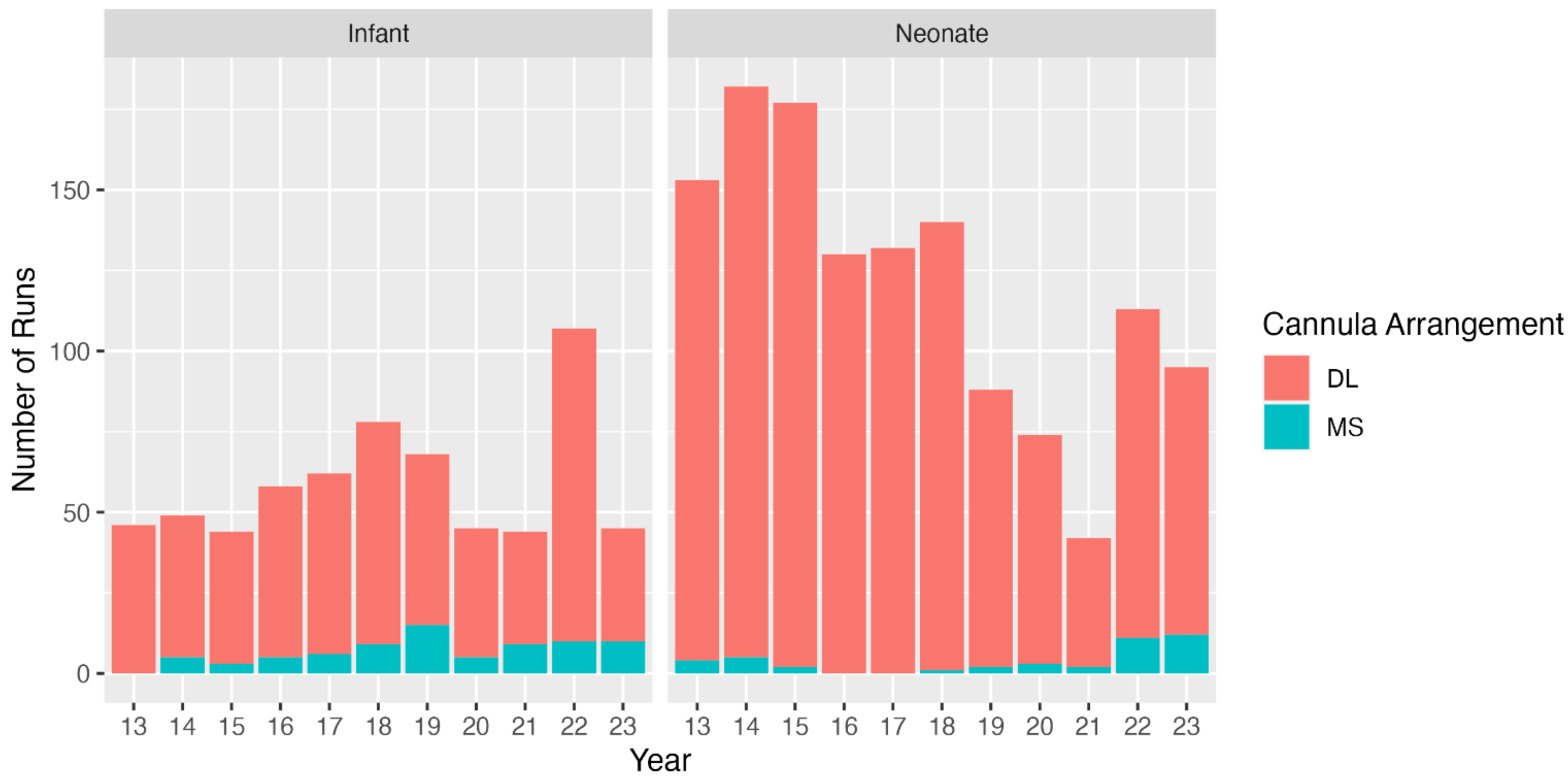

Figure S3. Trajectories over time for SaO_2 and ECMO flows, by age and cannula group.

Trajectory over time, estimated from the hierarchical linear mixed-effects models. Markers represent estimated marginal means, error bars 95% confidence intervals

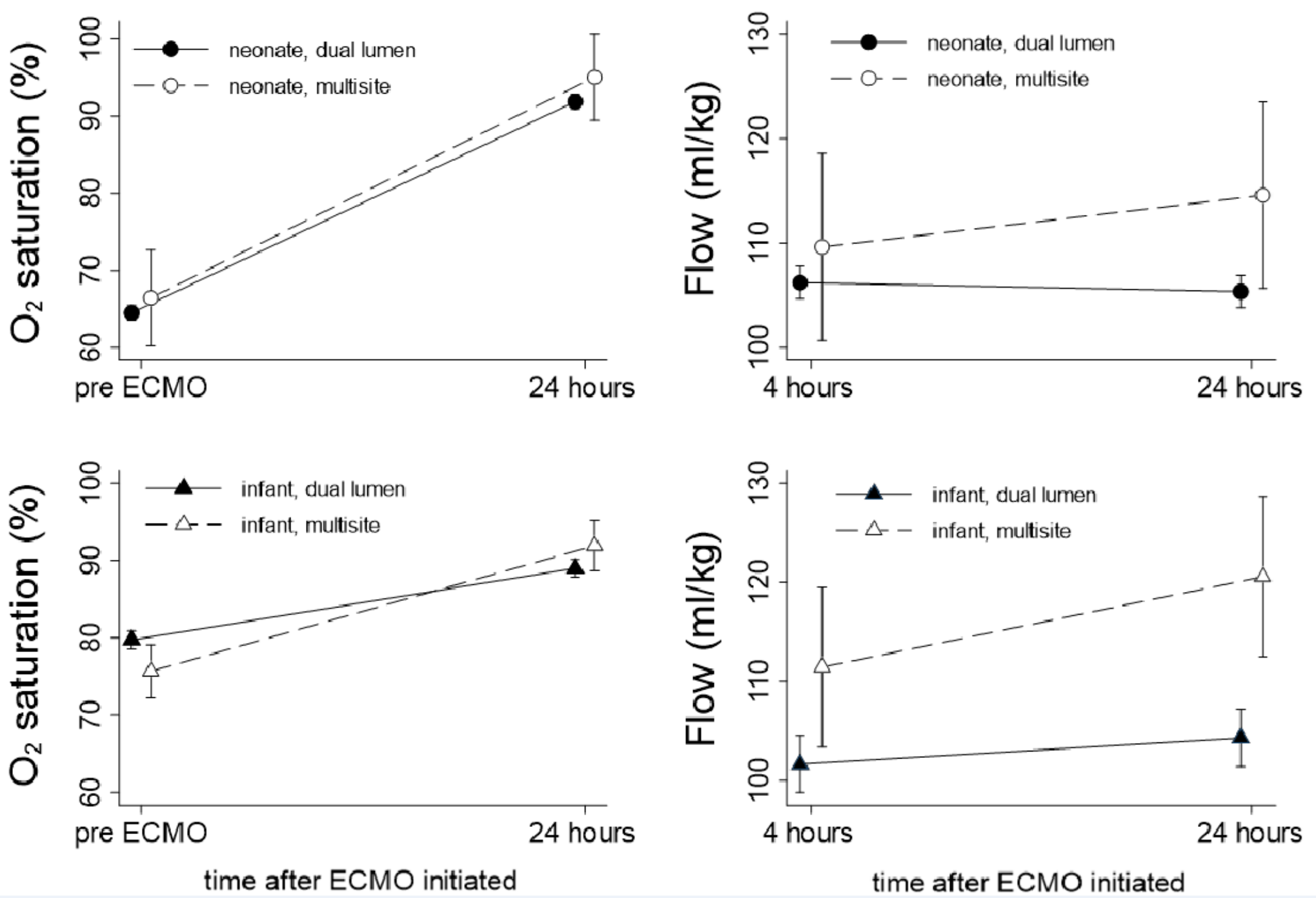

Table S1 Neonatal Access and Return Cannula sizes

Table showing pairs of cannulas used for neonates receiving VVMS. Thirty-four patients had cannula size data for both cannulas.

Access Cannula	Return cannula		
	7-8- Fr	9-10- Fr	11-12-Fr
7-8- Fr	5	2	0
9-10- Fr	9	2	2
11-12- Fr	8	3	0
13-14- Fr	2	1	0

Table S2 Multivariable logistic regression models examining factors associated with in hospital mortality. Missing data were accounted for using multiple imputation.

Neonates (n = 1445)				
**Variable**	**% missing**	**Odds Ratio**	**95% CI**	**P value**
VVMS	0	1.06	(0.49 to 2.30)	0.88
**Weight (kg)**	**0**	**0.65**	**(0.50 to 0.85)**	**0.001**
Cardiac arrest	1.2	1.46	0.87 to 2.43)	0.15
pH*	2.3	1.05	(0.96 to 1.15)	0.26
Oxygenation Index**	16.8	1.04	(0.99 to 1.08)	0.09
**Gestational age**	**5.6**	**0.83**	**(0.77 to 0.90)**	**<0.001**
**Diaphragmatic Hernia**	**0**	**3.20**	**(2.30 to 4.44)**	**<0.001**
**Infants (n = 680)**				
**Variable**	**% missing**	**Odds Ratio**	**95% CI**	**P value**
VVMS	0	1.05	(0.60 to 1.81)	0.87
**Weight (kg)**	**0**	**0.90**	**(0.82 to 0.98)**	**0.02**
Cardiac arrest	0.9	1.21	(0.65 to 2.24)	0.55
pH*	7.9	0.99	(0.89 to 1.10)	0.85
**Oxygenation Index****	**30.9**	**1.18**	**(1.08 to 1.30)**	**<0.001**

*odd ratio per 0.1 units increase pH, ** odds ratio per 10 units increase in oxygenation index.
